# The Efficacy of Adenoidectomy on Otitis Media with Effusion According to the Age of Child

**DOI:** 10.1007/s00405-025-09750-w

**Published:** 2025-10-19

**Authors:** Tomas Valenta, Lukas Skoloudik, Kristyna Nema, Jiri Haviger, Viktor Chrobok, Miroslav Vesely, Bretislav Gal

**Affiliations:** 1 Department of Otorhinolaryngology and Head and Neck Surgery, Faculty of Medicine in Hradec Kralove, Charles University, Hradec Králové, Czech Republic; 2 Department of Otorhinolaryngology and Head and Neck Surgery, University Hospital Hradec Kralove, Hradec Králové, Czech Republic; 3 Department of Otorhinolaryngology and Head and Neck Surgery, St. Anne’s University Hospital, Brno, Czech Republic; 4 Department of Otorhinolaryngology and Head and Neck Surgery, Faculty of Medicine, Masaryk University, Brno, Czech Republic; 5 Department of Informatics and Quantitative Methods, Faculty of Informatics and Management, University of Hradec Kralove, Hradec Králové, Czech Republic; 6 Department of Military Internal Medicine and Military Hygiene, Military Faculty of Medicine, University of Defense, Brno, Czech Republic

**Keywords:** Otitis media with effusion, Adenoid size, Adenoidectomy, Myringotomy

## Abstract

**Aim:**

The study compares the efficacy of adenoidectomy and myringotomy on the treatment of otitis media with effusion (OME) in children depending on the size of adenoids (AV) and age.

**Material and methods:**

A total of 509 children with OME lasting at least 3 months, who underwent endoscopic adenoidectomy (AT) and myringotomy without the insertion of a ventilation tube in the years 2010–2019 were enrolled in the study. The efficacy of AT and myringotomy on OME was evaluated with regard to the size of the adenoids, their relationship to the tubal tori, the viscosity of middle ear effusion and the age of the child. To evaluate the effect of age, children were divided into two groups—up to 4 years and over 4 years.

**Results:**

The efficacy of AT and myringotomy in OME treatment was demonstrated in both groups. Within the entire group, a significantly greater benefit of AT and myringotomy was found in children with adenoids in contact with the tubal tori (grade B) or compressing them (grade C) (p = 0.039). Other parameters were not statistically significant. Neither the size of the adenoids relative to the choanae, nor the children age, nor the type of middle ear effusion (serous, mucous) had an effect on the treatment.

**Conclusion:**

AT and myringotomy is an effective surgical procedure in the treatment of OME not only in children older than 4 years, but also in younger children. The effect of the surgery is affected by the contact of adenoids to tubal tori.

## Introduction

Otitis media with effusion (OME) is a disease characterized with development of effusion in the middle ear cavity behind intact tympanic membrane lasting at least 3 months. Patients present with conductive hearing loss, although, especially in children, the disease is often inapparent. It is one of the most frequent childhood diseases and could have negative effect on speech and intellect development. The prevalence rate in 5-year-olds is 15–40%. In most cases the disease is self-limited, however 30–40% children suffer from recurrent disease and in about 5–10% of children, the disease lasts more than 1 year. In adults, OME is scarce and could be one of initial signs of nasopharyngeal carcinoma [1]. Dysfunctional Eustachian tube (ET) is still deemed as ‘traditional’ factor in OME pathogenesis, however ‘new factors’ as viral infections, allergies, immunologic and microbial disbalances emerged in recent past [2–4].

Recommendations for managing OME differ worldwide. Due to OME being a self-limited disease in the first 3 to 6 months watch-and-wait approach with otomicroscopy and hearing evaluation is preferred by most physicians [1, 4, 5]. If the conservative approach fails, surgical treatment is indicated. The American Agency for Health Care Policy and Research states in its 1994 guideline ventilation tube (VT) insertion as the golden standard in OME surgical treatment. Adenoidectomy (AT) is recommended only in case of recurrent nasopharyngitis and nasal obstruction. The American Academy of Otolaryngology-Head and Neck Surgery (AAO-HNS) in its revised 2016 guideline recommends VT insertion for children up to 4 years of age. For older children the guideline recommends VT insertion with or without AT, or AT with myringotomy without VT insertion [1, 4]. Our study published in 2018 proved that AT with myringotomy without VT insertion has significant effect in managing OME and leads to long-lasting hearing improvement. However, this study did not take age of child into consideration [1]. The aim of the current study is to reevaluate the effect of such treatment according to the age of child.

## Material and methods

### Participants

A total of 1180 children with OME underwent surgical treatment consisting of AT with myringotomy at the Department of ENT-HNS of the University Hospital Hradec Kralove in between 2010 and 2019. Inclusion criteria for enrollment into the study were age up to 18 years, history of OME lasting at least 3 months and endoscopic finding of adenoids. OME was defined as persistence of middle ear effusion (MEE) behind intact tympanic membrane without signs of acute otitis media. Persistence of OME was evaluated by otomicroscopy and tympanometry. All children underwent routine diagnostic evaluation of nasopharynx via flexible epipharyngoscopy. Tympanometric criterion of OME was presence of B or C2 type tympanometric curve.

Pure tone audiogram was acquired in older children and in those younger ones who were able to provide valid responses. But due to difficulties (or inability) of acquiring valid pure tone audiogram in very young children, audiometric criteria and outcome were not included in study.

Patients who underwent simultaneous VT insertion, or those with incomplete documentation or postoperative follow-up shorter than 12 months were removed from the study.

**Chart 1:**
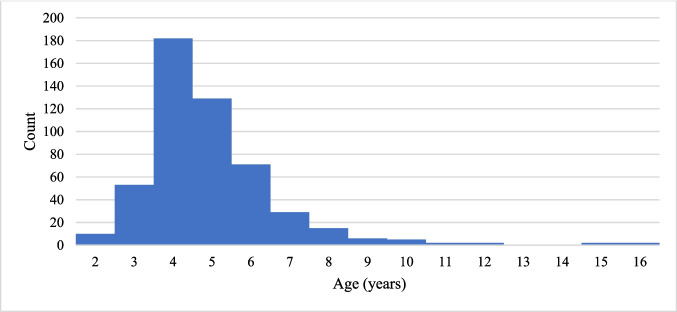
Histogram of age distribution

A total of 509 children (877 ears, 442 left ears, 435 right ears) aged 2–18 years, 246 up to 4 years of age and 263 older than 4 years were enrolled into study. Average age and median were 5 years. For age distribution of participants, see Chart 1.

### Surgical procedures

Medical history was obtained for each patient and tympanometry, eventually standard pure tone audiometry was performed on admission. Tympanometric curves were evaluated as follows: Type A curve with tympanometric width (TW) ± 100 daPa; type C curve with TW < −100 daPa; type B was flat curve.

Surgical procedures (endoscopic adenoidectomy with myringotomy with MEE aspiration) were carried out in general anesthesia. Myringotomy was performed uni- or bilaterally according to preoperative audiometric finding.

### Outcome measures

Adenoid size was evaluated during the surgery by performing surgeon using the Sychra’s system (Table 1) which combines evaluating adenoid size according to obstruction of choanae and to obstruction of Eustachian tubes orifices. A rigid endoscope with a diameter of 4 mm, a length of 18 cm and a viewing angle of 70 degrees was used in all patients.

**Table 1:** Adenoid size evaluation, Sychra’s system [6]

Adenoid size according to obstruction of choanae	Grade I – adenoids obstruct less than 1/3 of vertical portion of choanae
	Grade II – adenoids obstruct up to 2/3 of vertical portion of choanae
	Grade III – adenoids obstruct more than 2/3 of vertical portion of choanae
Adenoid size according to obstruction of Eustachian tubes orifices	Grade A – adenoids without contact with ET orifices
	Grade B – adenoids in contact with ET orifices, but do not obstruct them
	Grade C – adenoids completely obstructing ET orifices

For evaluating the efficacy of said procedure according to the age of child, patients were divided into 2 age groups – children up to 4 years of age and older children.

Criterion for successful procedure was a normal otoscopy finding (position and mobility of tympanic membrane) and change in tympanometric curve into type C1 or A within 12 months after the surgery.

Acquired data were evaluated by certified statistician.

### Statistical analysis

Statistical analysis of success rates was conducted using proportion-based methods. Confidence intervals for success probabilities and their deviations were calculated using proportion confidence intervals. Between-group comparisons were performed using two-sided proportion z-tests with statistical significance set at α = 0.05.

### Software statement

All statistical analyses were performed using Python (version 3.10.15) with packages Pandas (version 2.1.4), Pingouin (version: 0.5.5) and Statsmodels (version 0.14.4), specifically utilizing the *proportions_ztest* and *proportion_confint* functions from the *statsmodels.stats.proportion* module.

## Results

A total of 877 myringotomies were performed. MEE was found in 849 ears (427 right-sided, 422 left-sided). In the age group up to 4 years, myringotomy was performed in 432 cases (216 right ears, 216 left ears). In the age group older than 4 ears, myringotomy was performed in 445 cases (226 right ears, 219 left ears).

In the whole study group, AT with myringotomy led to OME healing up in 82% of patients. Out of the total count of 849 ears, surgical procedure was effective in 87% of ears.

Of possible surgical complications, we had 3 cases of postoperative hemorrhage (0.34%) out of which 2 required surgical revision. No other complications, such as hemotympanum, postoperative otorrhea, velopharyngeal insufficiency or Grisel’s syndrome were observed.

### Influence of adenoid size on the postoperative outcome

AT with myringotomy was more effective in children with grade B and C adenoids (p = 0.039). In children with grade A adenoids, procedure proved less effective. No significant difference in outcome was observed when evaluating adenoids according to obstruction of choanae (Grade I-III) (Table 2).

**Table 2:** Adenoid size and postoperative tympanogram outcomes in the total study sample: two-tailed proportion z-test (*n* = 877)

Whole study group, *n* = 877						Proportion test	
Variable	Value	Sucess	Sample	Prob	CI(prob)	Z	*p*-value
All		727	877	0.829	(0.804, 0.854)		
Adenoids size I-III	I	56	63	0.889	(0.811, 0.966)	1.311	0.190
	II + III	671	814	0.824	(0.798, 0.850)		
Adenoids size A-C	A	33	46	0.717	(0.587, 0.848)	−2.065	0.039
	B + C	694	831	0.835	(0.810, 0.860)		

### Influence of the character of middle ear effusion on the postoperative outcome

Mucous MEE was more frequent (61%), effect of surgical treatment was observed in 83% of all patients. Serous MEE was less frequent (39%), effect of surgical treatment was observed in 85% of all patients. We did not prove that character of MEE would affect the outcome of surgical treatment (Table 3). Viscosity of MEE was not affected by the age of child. In both subgroups, incidence of mucous (or serous) effusion was similar and we did not find significant statistical difference (Tables 4 and 5).

**Table 3:** Middle ear effusion (MEE) viscosity and postoperative tympanogram outcomes in the total study sample: two-tailed proportion z-test (*n* = 877)

Whole study group, n = 877						Proportion test	
Variable	Value	Sucess	Sample	Prob	CI(prob)	Z	*p*-value
All		727	877	0.829	(0.804, 0.854)		
MEE viscosity	Mucous	425	518	0.820	(0.787, 0.854)	−0.731	0.465
	Serous	278	331	0.840	(0.800, 0.879)

**Table 4:** Middle ear effusion (MEE) viscosity and postoperative tympanogram outcomes in the subgroup up to 4 years of age: two-tailed proportion z-test (*n* = 432)

Subsample age < = 4, *n* = 432							
Variable	Value	Sucess	Sample	Prob	CI(prob)	Z	*p*-value
MEE viscosity	Mucous	212	266	0.797	(0.749, 0.845)	−0.528	0.598
	Serous	126	154	0.818	(0.757, 0.879)

**Table 5:** Middle ear effusion (MEE) viscosity and postoperative tympanogram outcomes in the subgroup above 4 years of age: two-tailed proportion z-test (*n* = 445)

Subsample age > 4, *n* = 445							
variable	value	sucess	sample	prob	CI(prob)	Z	*p*-value
all		378	445	0.849	(0.816, 0.883)		
MEE viscosity	mucous	213	252	0.845	(0.801, 0.890)	−0.387	0.699
	serous	152	177	0.859	(0.807, 0.910)		

### Influence of the age of child on the postoperative outcome

#### Influence of the age of the child on the middle ear effusion viscosity

**Chart 2:**
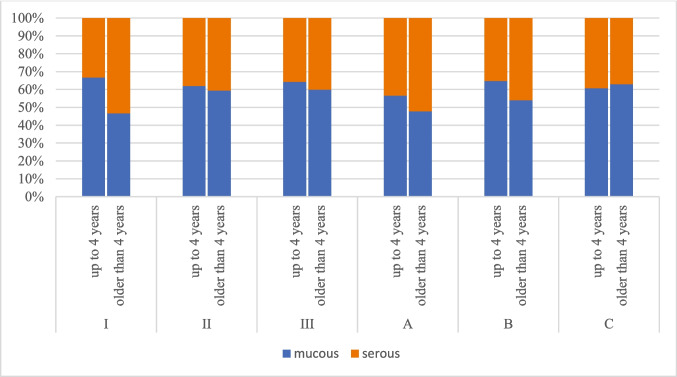
Middle ear effusion viscosity and adenoid size and age of child

In the age subgroup up to 4 years of age, MEE was found in 420 ears (210 right, 210 left), in 12 ears (6 right, 6 left) no MEE was found. Mucous effusion was more frequent (266 ears, 63%). Size of the adenoids did not have significant effect on MEE viscosity. The relationship between the age of child, the size of the adenoids and the nature of the middle ear effusion is shown in Chart 2.

In the age subgroup above 4 years of age, MEE was found in 429 ears (217 right, 212 left), no MEE was found in 16 ears (9 right, 7 left). Similarly, in this subgroup, mucous effusion was more frequent (252 ears, 59%). Size of the adenoids did not have significant effect on MEE viscosity. The relationship between the age of child, the size of the adenoids and the nature of the middle ear effusion is shown in Chart 2. Comparing both subgroups, we did not find significant difference in MEE viscosity between subgroups.

#### Influence of the age of child on the adenoid size

**Chart 3:**
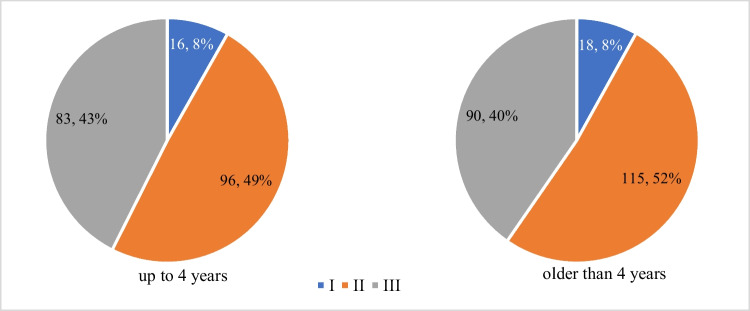
Adenoid size according to obstruction of choanae

**Chart 4:**
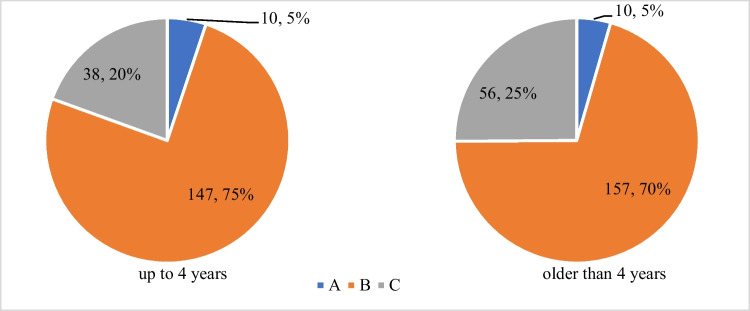
Adenoid size according to obstruction of Eustachian tubes orifices

Adenoids did not differ in size between both subgroups, the most frequent size was IIB. The distribution of individual adenoids sizes in both subgroups is shown in Charts 3 and 4. No statistically significant difference was found.

#### Influence of the age of child on efficacy of adenoidectomy and myringotomy

**Chart 5:**
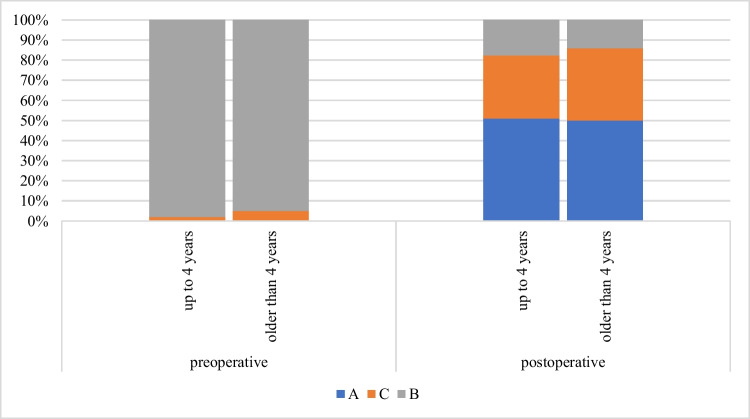
Tympanometry before and after surgery

AT with myringotomy proved to be effective in 706 (82%) ears. In children up to 4 years of age, surgery was effective in 343 (81%) ears, in children above 4 years of age in 363 (86%) of ears (Chart 5). We did not prove statistically significant difference between both subgroups.

Tables 6 and 7 summarize efficacy of surgical treatment according to age of child and size of adenoids.

**Table 6:** Adenoid size and postoperative tympanogram outcomes in the subgroup up to 4 years of age: two-tailed proportion z-test (*n* = 432)

Subsample age < = 4, n = 432							
Variable	Value	Sucess	Sample	Prob	CI(prob)	Z	P-value
All		349	432	0.808	(0.771, 0.845)		
Adenoids size I-III	I	28	32	0.875	(0.760, 0.990)	1.002	0.316
	II + III	321	400	0.802	(0.763, 0.842)		
Adenoids size A-C	A	16	23	0.696	(0.508, 0.884)	−1.404	0.160
	B + C	333	409	0.814	(0.776, 0.852)

**Table 7:** Adenoid size and postoperative tympanogram outcomes in the subgroup above 4 years of age: two-tailed proportion z-test (*n* = 445)

Subsample age > 4, *n* = 445							
Variable	Value	Sucess	Sample	Prob	CI(prob)	Z	*p*-value
All		378	445	0.849	(0.816, 0.883)		
Adenoids size I-III	I	28	31	0.903	(0.799, 1.000)	0.868	0.385
	II + III	350	414	0.845	(0.811, 0.880)		
Adenoids size A-C	A	17	23	0.739	(0.560, 0.919)	−1.519	0.129
	B + C	361	422	0.855	(0.822, 0.889)		

### Influence of the gender of child on the postoperative outcome

Comparing males and females we did not find difference of postoperative outcomes between both genders in the whole study groups as well as both age subgroups (Tables 8, 9 and 10).

**Table 8:** Gender and postoperative tympanogram outcomes in the whole study group: two-tailed proportion z-test (n = 877)

Whole study group, *n* = 877						Proportion test	
Variable	Value	Sucess	Sample	Prob	CI(prob)	Z	*p*-value
All		727	877	0.829	(0.804, 0.854)		
Gender	male	422	519	0.818	(0.785, 0.851)	−1.047	0.295
	female	305	361	0.845	(0.808, 0.882)

**Table 9:** Gender and postoperative tympanogram outcomes in the subgroup up to 4 years of age: two-tailed proportion z-test (*n* = 432)

Subsample age < = 4, *n* = 432							
Variable	Value	Sucess	Sample	Prob	CI(prob)	Z	*p*-value
All		349	432	0.808	(0.771, 0.845)		
Gender	Male	259	208	0.803	(0.755, 0.852)	−0.309	0.758
	Female	141	173	0.815	(0.757, 0.873)

**Table 10:** Gender and postoperative tympanogram outcomes in the subgroup above 4 years of age: two-tailed proportion z-test (*n* = 445)

Subsample age > 4, *n* = 445							
Variable	Value	Sucess	Sample	Prob	CI(prob)	Z	*p*-value
All		378	445	0.849	(0.816, 0.883)		
Gender	Male	214	257	0.833	(0.787, 0.878)	−1.155	0.248
	Female	164	188	0.872	(0.825, 0.920)		

## Discussion

The AAO-HNS guideline states different surgical approach in children with OME up to 4 years of age and older [4]. In younger children, authors recommend VT insertion as the method of choice. AT is recommended only in case of nasal obstruction or chronic adenoiditis. Authors state that AT in such young children is not justified with regard on inconsistent and scarce studies and possible surgical complications [4].

The most feared complication of AT, by both surgeon and parent, is postoperative hemorrhage. The incidence, however, is rare, studies state the rate of 0.28 to 1.32% [7–10]. In our group of patients, we had 3 cases of postoperative hemorrhage (0.34%) out of which 2 required surgical revision.

Other possible complications, such as velopharyngeal insufficiency, Grisel’s syndrome or nasopharyngeal stenosis are even rarer. Moreover, velopharyngeal insufficiency tends to be self-limited with complete restoration in 5 months after surgery in most cases [11–14]. Anesthesiologic risks are comparable with those in VT insertion. In our studied population, we did not encounter any of those.

Meta-analysis of 134 studies on VT insertion states the risk of intermittent otorrhea in 26% of cases, dislocation of VT into the middle ear cavity in 0.5% of cases, long-term sequelae such as tympanosclerosis in 32% of cases, focal tympanic membrane atrophy in 25% of cases, retraction pocket in 3.1% of cases, persistent tympanic membrane perforation in 2.2% of cases and secondary cholesteatoma in 0.7% of cases [15].

Eustachian tube dysfunction by mechanical obstruction by its pharyngeal orifice is still deemed as ‘traditional’ factor in OME development [6]. Moreover, there is a presence of biofilm on the adenoid surface which contributes to the development of chronic adenoiditis and immunologic changes that further impact Eustachian tube function [2, 3, 16, 17]. This represents rationale for implementing of AT into the management of OME in children younger than 4 years of age.

In older children, 2016 AAO-HNS guideline offers surgeon several surgical methods – VT insertion alone, VT accompanied with AT, AT alone or AT with myringotomy. Authors state that possibility to choose from at least 3 surgical approaches enable better cooperation between parent and surgeon. Authors explicitly state comparable results of AT alone and AT with VT insertion. Furthermore, authors assume the improvement of nasopharyngeal microenvironment due to removing inflamed adenoids. This compensates possible surgical complications of AT [4].

Our 2018 study proved AT with myringotomy to be effective in treatment of 87% of children with OME. Other recent studies yielded similar results [18, 19]. In addition, the benefit of AT has increased with the introduction of endoscopic control, which allows for proper curettage and reduces the likelihood of leaving residues [20], which can occur in up to 45% of cases with ‘classic’ AT according to Bross-Soriano [21]. VT insertion alone, without AT, leads to hearing improvement immediately after surgery and simultaneously eliminates potential surgical risks of AT, however the long-term effect decreases with time as the VT is removed or spontaneously eliminated [22–24].

In our current study, we demonstrated the success of AT in the treatment of OME not only in children older than 4 years, but also in younger children, with the procedure benefiting mainly in children with adenoids reaching or compressing the tubal tori (grades B and C). The size of the adenoids in relation to the choanae (grades I-III) is not a significant factor. Similar results were also obtained by Maw and Wright [25, 26]. In contrast, Paradise and Gates did not demonstrate a relationship between AV volume (size) and postoperative outcome (healing of OME) [27, 28].

The character of the middle ear effusion can be serous or mucous. The formation of mucous secretions implies a longer duration of the effusion, changes in the epithelium of the middle ear cavity and an increase in secretory glands. The mucous type of effusion is therefore often considered to be more prognostically serious [1]. In our study, we did not find that children with the mucous type of effusion had a significantly worse probability of its disappearance after the above-mentioned therapy. When comparing the two age groups, we did not observe a difference in the nature of the middle ear effusion.

Our study did not demonstrate a significant difference in the size of adenoids, the nature of middle ear effusion, and the effect of adenoidectomy on the healing of OME in children aged 2–4 years compared to older children. Also, potential complications of endoscopic adenoidectomy are rare, including postoperative bleeding. Therefore, we see no reason to recommend different treatment for OME in children aged 2–4 years and older children.

## Conclusion

According to our study, adenoidectomy with myringotomy is effective in the treatment of OME not only in children older than 4 years, but also in younger children. This treatment is more effective in children with adenoids reaching or compressing the tubal tori (size B and C). Neither the size of the adenoids relative to the choanae, nor the viscosity of middle ear effusion (serous, mucous) had an effect on the surgery treatment.

Thus, based on this, we recommend adenoidectomy with myringotomy also for children under 4 years of age. The procedure is both effective and safe. Furthermore, it allows to avoid both short- and long-term sequelae of myringostomy with ventilation tubes.

## Study limitations

The study proved a similar effect of adenoidectomy with myringotomy in children over 4 years of age as in younger children, but we did not compare these results with children who underwent standalone VT placement, or adenoidectomy with VT placement, as a primary surgical modality. Further studies are needed to compare these different surgical approaches.
